# Retinoic Acid Receptor γ Is a Ligand-Activated Gatekeeper to Stem Cell Developmental Progression

**DOI:** 10.3390/ijms27167160

**Published:** 2026-08-11

**Authors:** William Eustace Basil Johnson, Caitlin McQueen, Geoffrey Brown

**Affiliations:** 1 Chester Medical School, University of Chester, Chester CH1 4B1, UK; 2 Norwich Medical School, Faculty of Medicine and Health Sciences, University of East Anglia, Norwich Research Park, Norwich NR4 7TL, UK; caitlin.mcqueen@uea.ac.uk; 3 Department of Biomedical Sciences, College of Medicine and Health, University of Birmingham, Edgbaston, Birmingham B15 2TT, UK

**Keywords:** retinoic acid receptors, embryogenesis, normal stem cells, cancer stem cells, cell differentiation

## Abstract

RARγ is expressed during embryogenesis by stem/primitive progenitor cells, indicating a role in controlling their development. Supporting this view is that a physiological level of 10 nM of the RARγ agonist AGN205327 blocked stem/progenitor cell differentiation during zebrafish embryogenesis, mouse gastruloid development, and adult chondro- and osteogenesis. Similarly, transgene expression of RARγ or the use of the RARγ agonist CD437 enhanced the generation of induced pluripotent stem cells (iPSCs) from human and mouse somatic cells. RARγ regulates many events that control the behavior of stem/progenitor cells regarding whether they develop to give rise to mature cells. RARγ positively regulates the expressions of NOTCH ligands and their receptors, transforming growth factors (TGFs), and molecules pertaining to cell identity, extracellular matrix communication, and all-trans retinoic acid synthesis and catabolism. The genes that are repressed by RARγ include RARγ, PPARγ, and RXRα. RARγ integrates into Wnt/β-catenin and TGFβ signaling by acting as a co-factor to the gene co-activator β-catenin and transcription factor Smad3, respectively. Within the cytoplasm, RARγ regulates Akt/NF-κB signaling. As a model, we propose that ATRA ligand-activated RARγ acts as a gatekeeper to stem cell developmental progression during embryogenesis and that this role extends to stem/progenitor cell homeostasis within adult tissues.

## 1. Introduction

Retinoic acid receptors (RARs) bind to the *cis*-acting response elements of target genes as a heterodimer with the retinoid X receptor. When all-trans retinoic acid (ATRA), the most active metabolite of vitamin A, is absent, target gene expression is repressed via the recruitment of co-repressors to RARs. ATRA binding to RARs leads to co-repressor release, the binding of co-activators, and gene expression [1,2]. ATRA is an ancient signaling molecule with a single ancestral RAR existing in bilaterian animals such as the annelid *Platynereis dumerilii* [3]. To activate gene transcription, ATRA bound with a low (μM) affinity to the RAR via a pocket different from that used by vertebrate RARs. *Platynereis dumerilii* larvae responded to exogenous ATRA in the medial neuroectoderm, leading to the view that RAR-mediated signaling is a spatial trigger for neurogenesis and axon outgrowth. The ancestral RAR was RARα-like from studies of the pocket structures of RAR paralogs. The three subtypes RARα, β, and γ emerged in jawed vertebrates (gnathostomes) after their split from jawless vertebrates (cyclostomes, e.g., lamprey). Vertebrate RARα, β, and γ have a high (nM) affinity for ATRA, and their ligand-binding pockets have diverged to accommodate substantially broad roles. The amino acid sequence of the N-terminus ligand-independent transcriptional activation domain (AF-1) varies among RARs, which may underlie subtype-specific biological roles. Conservation of the RARγ amino acid sequence across vertebrates, e.g., human and mouse, is remarkably high and much more than that for RARα, β, and γ within a species, indicating a unique role for RARγ [4].

Indeed, RARs are indispensable for vertebrate embryonic development [5] because they regulate fundamental cell processes including survival, proliferation, differentiation, and migration. Some 160 years ago, Virchow postulated that development goes awry in cancer. Advances in molecular biology have highlighted drivers that are common to embryogenesis and carcinogenesis, whereby the events that regulate normal developmental are dysregulated in cancer [reviewed in [6]]. RARγ is a major focus of attention because it is oncogenic in many cancers. They include some cases of acute myeloid leukemia [7], cholangiocarcinoma [8] and colorectal [9], gastric [10], head and neck [11], hepatocellular [12], ovarian [13], pancreatic [14], prostate [15], renal [16], and thyroid [17] cancers. This review examines the unique roles that have been attributed to RARγ, focusing on the findings for normal development whereby an understanding is also key to unlocking the role of RARγ in cancer.

## 2. RARγ Plays a Particular Role During Development

RAR isoforms substitute for one another because double knockout of RARs in mice was needed to mimic some of the developmental defects within embryos from dams that were rendered vitamin A-deficient [18,19]. However, isoforms play different roles: for example, they regulate ATRA metabolism because loss of RARγ by F9 embryonal carcinoma cells led to reduced metabolism, whereas loss of RARα led to increased ATRA metabolism as compared to wild-type cells [20]. Also, RARγ plays a dominant role in a context-dependent manner. RARα or RARγ mediated ATRA-induced differentiation of P19 embryonal carcinoma cells into various cell types, whereas only RARγ mediated the differentiation of F9 embryonal carcinoma cells into primitive endoderm [21]. From the use of RAR selective agonists, RARγ was seen to be functionally dominant regarding the development of striatopallidal medium spiny neurons from embryonal carcinoma cells [22]. Findings for the expression of the superfamily of nuclear hormone receptors within embryonic stem cells (ESCs) provided support to the core role of RARγ within ESCs. Undifferentiated human H1 and H9 ESCs, as well as mouse CMT1-1 ESCs, expressed mRNAs for 42 nuclear hormone receptors. RARγ was shared by humans and mice and was >10-fold higher than any other nuclear hormone receptor within mouse ESCs. RARγ mRNA markedly declined at day 1 when ESCs differentiated towards embryoid bodies [23].

Whereas RARα mRNA is expressed broadly by embryonic and adult cells, RARγ mRNA is restricted to stem/primitive progenitor cells. During mouse embryogenesis, the stem/progenitor cells that express RARγ include early mesoderm cells developed from the primitive streak, germ cell layers in the posterior embryo region (i.e., frontonasal and pharyngeal mesenchymal derivatives), all pre-cartilaginous mesenchymal condensations, primitive neural ectoderm and endoderm derivatives, epithelia when they had started to differentiate, whisker follicle root cells, and cells where teeth develop [24]. This pattern indicates that RARγ regulates morphogenesis, chondrogenesis, and squamous epithelial cell differentiation. During zebrafish embryogenesis, mRNAs for RARγa and RARγb paralogs were expressed by three streams of migrating cranial neural crest cells and cells in the anterior and tailbud regions [25]. Other workers reported RARγ expression in zebrafish by mesodermal and neural crest stem/progenitor cells in the head area, the lateral plate mesoderm, and the presomitic mesoderm of the tail bud [26]. Hematopoietic stem cells (HSCs) in the adult mouse are within lineage^−^, c-kit^+^, and Sca-1^+^ (LKS^+^) cells, and these cells selectively expressed RARγ1 [27]. Hence, HSCs and their immediate offspring express RARγ1, supporting a unique role of RARγ within HSCs.

It is important that whilst stem/primitive progenitor cells express both RARα and RARγ, RARγ can play a dominant role by virtue of its transactivation by sub nM ATRA. The affinities of RARs for ATRA are similar (ED_50_ values of 3–10 nM) [28], but the level of ATRA that transactivated RARγ and RARβ was 33-fold lower than that for RARα (~0.4 versus ~13 nM) [28,29]. This is highly relevant to the behavior of stem/primitive progenitor cells, which do not appear to synthesize ATRA because they lack the required synthetic enzymes and the specific cell surface receptor stimulated by retinoic acid 6 (STRA6 for all-trans retinol uptake) [30,31]. Nonetheless, cultures of undifferentiated mouse ESCs (a clone of D3 cells) contained ~1 nM ATRA (from fetal calf serum), which was enough to transactivate RARγ to influence gene expression. Treatment of ESCs with the pan-RAR inverse agonist BMS493 (for antagonism) decreased the basal expression of the *RARβ* and *Hox1* genes and increased the expression of *Foxd3* and *Otx2*, which are ATRA-repressed genes [32].

Findings for the generation of iPSCs from somatic cells highlighted RARγ as playing a unique role in the establishment and/or maintenance of naïve pluripotency [33,34,35]. Mouse embryonic fibroblast reprogramming was enhanced and shortened to 4 days by transgene expression of RARγ and the orphan nuclear receptor liver receptor homolog 1 (LRH-1) when the transcription factors Oct4, Sox2, c-Myc, and Klf4 were used to generate iPSCs. Removal of all-trans retinol from the medium or the use of an RARγ agonist confirmed the need for active RARγ, and the action of liver receptor homolog 1 was also dependent on liganded RARγ [33]. A dominant-negative form of *RARA* impeded reprogramming [34]. iPSCs were derived from human dermal fibroblasts by using agonists of RARγ (CD437) and LRH-1 (RJW101), inhibitors of glycogen synthase kinase and MEK1/2, and transgene expression of Oct4, Sox2, Klf4, L-Myc, and p53 [35].

RARγ is unique among RARs in localizing to the cytoplasm via its N-terminal A/B domain, as seen for confluent H460 lung cancer cells. By contrast, RARγ was predominantly nuclear for sub-confluent cells cultured serum-free and localized to the cytoplasm when serum-free cells were released from 24 h of serum starvation or treated with epidermal growth factor or platelet-derived growth factor. These effects were not seen for RARα. Cytoplasmic retention of RARγ was not dependent on nuclear export because leptomycin B inhibition of Crm-1-dependent nuclear export had no effect [36]. Localization to the cytoplasm may provide a critical regulatory function whereby it is poised to activate signaling cascades in response to ligand presence (discussed later in Section 4.3). Hence, the localization of RARγ is germane to whether RARγ is playing a genomic or non-genomic role within cells.

## 3. Agonism of RARγ Blocks Stem/Progenitor Cell Development

### 3.1. Findings from Studies of Embryogensis

Highly selective agonists and antagonists of RAR subtypes [37] were used to investigate the roles of RARs within zebrafish embryos. Synthetic agonists and antagonists encode a distinct ligand-binding domain (LBD) of their RAR subtype [38]. Antagonists fit into the LBD to prevent the adoption of an active conformation and even to favor co-repressor recruitment [reviewed in [39]]. Treatment of embryos at 4 h post-fertilization (hpf) with 10 nM of the RARγ agonist AGN205327, in the absence of exogenous ATRA, led to substantial disruption to tissue formation, which was not seen for the RARα agonist AGN195183 [40]. Defects included severe posterior truncation, fewer tail somites, a lack of the caudal and pectoral fins, craniofacial bones, and anterior neural ganglia, and cardiac edema. These effects correlate with the expression of RARγ paralogs. T-box transcription factor 5-positive (Tbx5) progenitor cells for pectoral fin formation were present in the agonist-treated fish and RARγ agonist washout experiments and changing to a medium containing an equimolar amount (10 nM) of the RARγ antagonist AGN205728 at 27 hpf restored fin development. Washout of 10 nM of the RARγ agonist or reversal of the action of the RARγ agonist, via the addition of the RARγ antagonist, significantly increased caudal fin length as compared to embryos treated with the RARγ agonist alone. The RARγ agonist also blocked caudal fin regrowth when transected at 2 days post-embryo fertilization and subsequent agonist washout or additional treatment with the RARγ antagonist one day after transection increased fin length.

RARγ2 is the major isoform that is expressed throughout the caudal axial progenitor domain of vertebrates. Activity in late-stage embryos indicated that RARγ2 plays a role in terminating elongation. Treatment of Xenopus embryos with the RARγ agonist NRX204647 at 100 nM or expression of a constitutively active VP16-RARγ2 led to diminished expression of caudal genes and premature termination of axis extension. The investigators concluded that RARγ2 became active due to the proximity of ATRA at the determination front of the caudal domain, which had led to apoptosis when the progenitor pool had become exhausted. In the absence of ATRA, RARγ2 repressed transcriptional activity to maintain the caudal progenitor cell pool and the presomitic mesoderm cells, which give rise to future somites and their derivatives. Conversely, RARγ2 facilitated somite differentiation when ATRA was present [41]. Inactive RARγ2 had either ensured the survival of the caudal progenitor cell pool and the presomitic mesoderm cells or allowed a primitive cell compartment to develop towards progenitors. During early mesoderm development and from knockdown studies, RARγ1 functioned as a transcriptional activator for mesodermal gene expression and stabilized this fate. RARγ1 was also required for somite boundaries and the differentiation of terminal skeletal muscle [42]. Hence, RARγ1 and RARγ2 play various roles during Xenopus embryogenesis to govern patterning in a spatial-, temporal-, and ligand-dependent manner.

ESC-derived mouse gastruloids recapitulate many aspects of early embryonic development [43]. They are generated by aggregating 200–300 ESCs, derived from the inner cell mass of the pre-implantation embryo, into spheroids. They are then pulsed with the Wnt/β catenin agonist CHIR99021 between 48 and 72 h, and after ~120 h, the spheroids have developed into “gastruloids” that had broken symmetry, formed 3 orthogonal axes, and undergone elongation [44,45,46]. RARγ mRNA was expressed in developing gastruloids predominantly within primitive tissues containing stem/progenitor cells, namely epiblasts, neuromesodermal progenitors, caudal epiblasts, as well as caudal mesoderm, rostral neuroectoderm, and primitive streak cells. The RARγ-positive cells expressed mRNAs for the development-associated transcription factors Nanog, Oct4, Sox2, and brachyury, but not for Aldh1a2 (for ATRA synthesis), Cyp26a1 (for ATRA catabolism), or Sox1 (for neural stem cell maintenance). It has been known for some time that treating CHIR99021-pulsed spheroids with 0.4–33 nM ATRA blocks elongation of mouse and human gastruloids [47]. Recently, it was shown that treatment of mouse gastruloids with 10 nM of the RARγ agonist AGN205327 was sufficient to block axial elongation, which was not seen for the RARα antagonist AGN195183. Examination of brachyury and Sox2 expressions confirmed that the RARγ agonist-treated gastruloids had failed to break symmetry. Brachyury immuno-positivity was localized to the posterior of control gastruloids that had elongated at 120 h, and Sox2 expression was often close by. For the RARγ agonist-treated spheroids that had remained as such, brachyury was present in clusters of cells throughout the spheroid, and Sox2 expression was also seen in groups of clustered cells [48]. Furthermore, this study showed that a blockage of gastruloid elongation by 10 nM ATRA was partially reversed by concurrent treatment with 100 nM of the RARγ antagonist AGN205728. As seen from the zebrafish studies, RARγ agonism had blocked the development of stem/progenitor cells.

### 3.2. Findings for Tisue-Specific Stem/Progenitor Cells

The findings for embryogenesis provided evidence that ligand-activated RARγ prevents stem/primitive progenitor cell development. Findings for tissue-specific stem/progenitor cells further support this view. RARγ is the major RAR isoform that is expressed in mouse growth plate chondrocytes, indicating a role in chondrogenesis and bone formation [49,50]. Treatment of micro-mass cultures of mesenchymal embryo limb cells obtained from day 11.5 embryos with 3–30 nM of the RARγ agonist NRX20467 blocked cartilaginous nodule formation. Ectopic bone mass formation was blocked when cultured mesenchymal cells were treated with the RARγ agonist and then injected into nude mice. The RARγ agonist also blocked heterotopic ossification in a transgenic model of fibrodysplasia ossificans progressiva [51]. Reduced chondrogenesis was seen when forelimbs from E12.5 mice were cultured for 6 days with the RARγ agonist BMS-18996 [52].

In hematopoiesis, HSCs were reduced ~3-fold and very primitive multilineage repopulating cells were profoundly decreased within the bone marrow of RARγ^−/−^ mice femurs. There was a corresponding increase in differentiated progenitor cells [27]. This was not seen for RARα^−/−^ mice. Active RARγ maintained HSCs because ATRA promoted their maintenance in vitro, and this potentiating effect was abrogated by the loss of RARγ. LSK^+^ cells from RARγ^+/+^ and RARγ^−/−^ mice also failed to show a competitive repopulating potential when cultured without ATRA. A more undifferentiated phenotype was exhibited by primitive hematopoietic precursors that had been transduced to overexpress RARγ1. Mature myeloid cells were increased in conditional RARγ^−/−^ mice and when wild-type mice were treated with the pan-RAR antagonist AGN194310 [53], indicating that an absence of the action of RARγ had allowed HSCs to develop.

## 4. The Role of RARγ Is Multifaceted, Involving Interaction with Other Transcriptional Regulators and Key Regulatory Signaling Pathways

### 4.1. RARγ Plays a Role Primarily Within the Nucleus

RARγ regulates hundreds of genes, either directly or indirectly, as seen for ATRA-induced differentiation of F9 ESCs [54]. Target genes have been identified within human oral squamous cell carcinoma cells whereby RARγ is a tumor suppressor. For RARγ knockout cells minus added ligands, mRNAs for NOTCH1, NOTCH3, the NOTCH ligands JAG2 and DLL1, genes pertaining to cell identity and extracellular matrix communication, as well as retinaldehyde reductase DHRS3, were reduced. The expressions of *RARG*, *PPARG*, and *RXRA* were at a higher level. These genes appear to be repressed by RARγ [55]. Other workers have reported that activation of RARγ following its ligation increased cell surface β1 integrin and augmented cellular adhesion [56] and inhibited expression of *PPARG* [57]. Within F9 embryonal carcinoma cells, RAR/RXR dimers bound to gene loci that bound ESRRB, KLF4, NANOG, NR5A2, POU5f1, SOX2, and TFCP2L1, indicating a role for RARs in regulating these stem cell pluripotency-associated transcription factors [58]. OCT4 plays an essential role in maintaining the state of mouse ESCs and directing differentiation. A subset of ATRA-responsive genes was co-occupied by RAR/RXR dimers and OCT4, and findings supported the view that OCT4 positively controls the level of RARγ [59].

**Figure 1:**
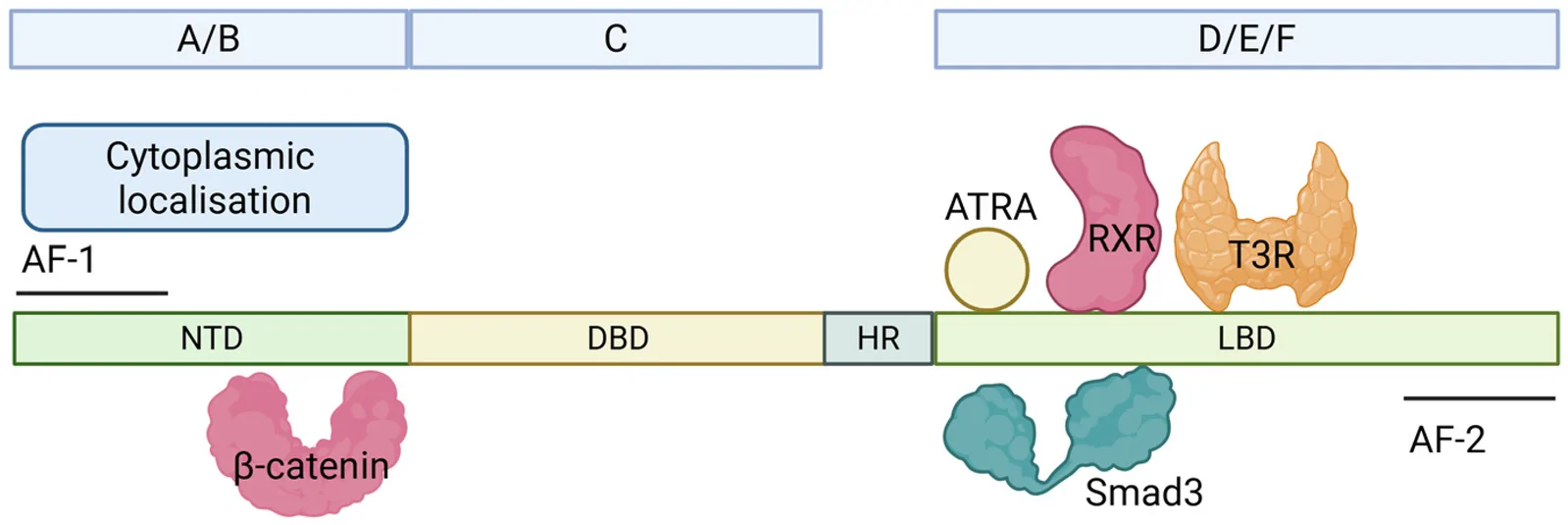
RARγ domains bind various molecules. RARγ domains include the N-terminal region (NTD), the DNA-binding domain (DBD), a hinge region (HR), the ligand (ATRA)-binding domain (LBD), and two activation function (AF) domains (AF-1 and AF-2). The figure shows the binding sites for all-trans retinoic acid (ATRA), the transcription factors retinoid X receptor (RXR) and Smad 3, and the transcriptional co-activator β-catenin. The cytoplasmic localization domain is also shown. Created in BioRender. Mcqueen, C. (2026) https://BioRender.com/b0i9755 (accessed on 12 July 2026).

RARγ regulation of RXRα is significant because RXRα forms a function dimer with multiple steroid hormone nuclear receptors. Additionally, RARγ dimerizes with thyroid hormone receptors, which are members of the nuclear hormone receptor superfamily. Figure 1 shows the domain structure of RARγ. The LBD of RARγ is the interface for the formation of a stable dimer with RXR, and a strong dimerization interface in helix 9 enables the interaction of RARγ with RXR in the absence of DNA [60,61]. RARs are efficient partners for thyroid hormone receptors by means of the LBD [62]. RARγ signaling also crosstalks with steroid hormone nuclear receptors [63], for example, to impact androgen signaling [64]. Hence, RARγ has a broad range of action.

### 4.2. RARγ Interacts with Smad3 and β-Catenin

For lung fibroblasts, RARγ interacted with Smad3, via the D/E/F sequence region that corresponds to the LBD [Figure 1] [65]. The influence of RARγ as a Smad3 co-factor was revealed from examining TGFβ/Smad3-mediated signaling as measured using a (CAGA)_9_-lux reporter. HepG2 cells were transfected to overexpress RARα, RARβ, or RARγ, with or without overexpression of Smad3. Smad3 resides in the cytoplasm as a latent protein that is translocated to the nucleus upon phosphorylation by the TGFβ receptor [66]. HepG2 cells were transfected with RARγ plus or minus the overexpression of Smad 3 and then treated with TGFβ. Antagonism of all RARs (using the pan-antagonist AGN194310) and specific antagonism of RARγ (AGN205728) enhanced reporting. Conversely, agonism of all RARs (AGN191183) and specific agonism of RARγ (AGN205327) decreased reporting. For cells transfected with RARα plus or minus Smad 3 and then treated with TGFβ, antagonism of all RARs and RARα (AGN196886) enhanced reporting, and agonism of all RARs and RARα (AGN195183) decreased reporting. Agonism of RARγ also decreased reporting for cells transfected with RARα [67]. From reporter experiments using lung fibroblasts that had been transfected with RARs, non-liganded RARγ was the most potent among RARs in enhancing cell responsiveness to TGFβ [65]. Hence, RARα and RARγ had enhanced or applied a brake to TGFβ responsiveness when antagonized or ligand-activated, respectively [67].

RARγ interacts with β-catenin, via its N-terminal AF-1 domain [Figure 1], as seen from co-immunoprecipitation studies using COS-7 kidney fibroblast-like cells transfected with hemagglutinin-tagged RARγ [68]. Non-liganded RARγ interreacted with β-catenin because complex formation was not seen for ATRA-treated cells. This study also examined the interaction between the Wnt/β-catenin and RAR pathways within neonatal mouse epiphyseal cartilage chondrocytes. From lymphoid enhancer factor/T-cell factor/β-catenin reporter and β-catenin nuclear accumulation studies, Wnt/β-catenin signaling increased when chondrocytes were treated with ATRA, in the presence or absence of exogenous Wnt3a. There was increased expression of Wnt proteins and receptors. For retinoid-free cultures, RARγ overexpression inhibited Wnt/β-catenin signaling and silencing of endogenous RARγ increased signaling, which was not seen for RARα or RARβ silencing. Two hybrid assays were used to examine the influence of RARγ on the association of β-catenin with the lymphoid enhancer factor/T-cell factor. Non-liganded RARγ, from overexpression within ATRA-free cells, dissociated β-catenin from the lymphoid enhancer factor/T-cell factor. The investigators concluded that liganded RARγ enhances Wnt/β-catenin signaling as seen for ATRA-stimulated expression of Wnt pathway components. Conversely, unliganded RARγ inhibited Wnt/β-catenin signaling because RARγ binding to β-catenin within the nucleus had prevented β-catenin from interacting with the lymphoid enhancer factor/T-cell factor.

### 4.3. Non-Genomic Actions Within the Cytoplasm

As mentioned in Section 2, RARγ localizes to the cell cytoplasm, and studies aiming to identify a role within the cytoplasm have focused on cancer cells whereby RARγ is an oncogene. It resides mainly in the cytoplasm of hepatocellular cancer, cholangiocarcinoma, and colorectal cancer cells [8,9,12]. Non-genomic actions of RARγ include activation of the Akt/NF-κB signaling in hepatocellular cancer [12], Akt/NF-κB and Wnt/β-catenin signaling in cholangiocarcinoma [8], and Wnt/β-catenin signaling in colorectal [9] and gastric cancer cells [10]. It is important to distinguish between findings for cancer cells and from developmental studies because, for cancer cells, there is likely to be cooperativity between overexpression of RARγ and other oncogenes, as seen by some cancers (see later). Indeed, in non-cancerous satellite cells, which are multipotent muscle stem cells, RARγ inactivated Akt. RARγ stimulated the genes that are responsible for Akt dephosphorylation. This maintained satellite cell quiescence, whereby the initiation of overall protein translation was inhibited. The cells were released from quiescence by alleviation of ATRA signaling [69]. RAR signaling also regulates dormancy of HSCs by restricting protein translation [70]. For mouse embryonic fibroblasts, RARγ has been reported to play a role in the formation of the Riptosome complex within the cytoplasm, which is required for necroptosis [71].

## 5. Longstanding Lessons from Studies of Avian and Mouse Limb Bud Development

For many years, limb bud development in chickens has provided a key model for studies of vertebrate morphogenesis. RARα and RARγ mRNAs are present in the interdigital regions of day 9 chick hindlimbs [72]. The findings for mouse hindlimbs are different, with RARα and RARγ mRNAs seen to be uniformly distributed in the limb bud at day 10 post-coitum. Cellular retinoic acid-binding protein (CRABP) transcripts showed a graded proximodistal distribution, indicating that its differential expression may establish the ATRA morphogenetic field. At later stages, RARγ mRNA was specific to the cartilage cell lineage and differentiating skin.

ATRA signaling is crucial to initial limb positioning and the establishment of major axes. At stage HH20/21, the posterior half of the chicken limb bud synthesizes ATRA at a higher rate than the anterior half, giving rise to a gradient [73]. A signal from the paraxial mesoderm is needed for limb bud formation because placing an impermeable barrier between the somites and the adjacent lateral plate mesoderm led to forelimb and hindlimb absence [74,75]. ATRA activation of RARs had been prevented because limb formation was also blocked when beads soaked in 2.5–5.0 mg/mL of the pan-RAR inverse agonist BMS493 (for antagonism) were applied to the bud. When a barrier to ATRA was applied early, brachyury mRNA expression in the lateral-plate mesoderm was downregulated and was rescued by the application of an ATRA-soaked bead. ATRA, therefore, regulates brachyury expression at the limb induction stage. Administering disulphiram to chicken embryos to prevent ATRA synthesis prior to limb bud outgrowth also largely abolished limb formation. This was rescued by implantation of an ATRA-soaked bead.

The expression of fibroblast growth factor (FGF)10 mRNA, in the lateral plate mesoderm, and of FGF8 mRNA, in the overlying ectoderm, were not initiated when limb formation was blocked by using pan-RAR inverse agonist-soaked beads. Expression of Tbx5 (for forelimb development) or Tbx4 (for hindlimb development) mRNAs was seen to be normal. Hence, FGFs are essential to limb formation, and FGF10 expression has been shown to be related to RAR signaling and shown to act cooperatively with β-catenin/lymphoid enhancer factor/T-cell factor and Hox factors [76]. Disulphiram, to prevent ATRA synthesis, led to an absence of sonic hedgehog (*Shh*) expression (a determinant of cell fate), despite the normal expression of *FGF8* [75,77]. Administration of disulphiram at later stages reduced ATRA synthesis, leading to a loss of expression of *Shh* and *FGF4*, and *FGF8* expression, as seen to be reduced at an earlier stage, was unaffected. A feedback mechanism controls limb initiation, with ATRA regulating FGF expression in the lateral-plate mesoderm and the overlying ectoderm.

Wnt signaling plays a central role by regulating initiation, apical ectodermal ridge (AER) formation, axis specification, stem/progenitor cell maintenance and distal morphogenesis through canonical β-catenin-dependent and non-canonical pathways [78]. Wnt7a signaling from the dorsal ectoderm specifies dorsal limb identity through the induction of Lmx1b. Signaling contributes also indirectly to the maintenance of *Shh* expression within the zone of polarizing activity and regulation of Shh pathway genes [79,80]. Non-canonical Wnt5a signaling regulates distal outgrowth, oriented cell behaviors and tissue polarity during limb elongation through planar cell polarity pathways [81].

Regarding ectoderm development, Wnt3/Wnt3a signaling activated β-catenin/TCF-mediated transcription of FGF8 within the AER. These events established the reciprocal FGF10–Wnt–FGF8 feedback loop that sustains limb bud outgrowth, and FGF signaling genes were downregulated when Wnt signaling was inhibited [80,82]. Bone morphogenic protein (BMP) signaling interacted with this regulatory circuit by restricting AER maintenance and promoting eventual ridge regression. BMP activity within the distal mesenchyme and ectoderm antagonizes prolonged FGF signaling and contributed to termination of proliferative outgrowth programs and limitation of posterior digit number [83,84]. BMPs are present in the distal mesenchyme and maintained their expression even when distal mesenchymal progenitors were transplanted to earlier-stage limb buds. BMPs regulate distal programming via cell-cycle parameters as demonstrated by implantation of noggin to grafted younger host cells. This resulted in a significantly lower level of cells in the G1-to-S phase of cell cycle than that seen for non-implantation controls [85].

The Wnt/FGF/ATRA signaling pathways also overlap to regulate distal mesenchymal progenitor competence. Primary mesenchymal cells remained undifferentiated when cultured in Wnt3a/FGF8/ATRA and the expression of early limb mesenchyme genes was maintained [86]. Other studies have shown that ATRA between FGFs and Wnts cooperatively maintains mesenchymal progenitors in an undifferentiated, proliferative state [87,88]. This alludes to a role for proximal signals, particularly ATRA, in maintaining stem/progenitor cells. ATRA is depleted from the chick wing bud by around the Hamburger–Hamilton (HH)20/21 stage whereby there is activation of an intrinsic distal program of gene expression. BMP signaling subsequently promotes transition away from this proliferative state by regulating cell-cycle kinetics and distal transcriptional timing programs [85,89,90]. The time of ATRA removal can alter the duration that *Shh* is intrinsically expressed in the polarizing region [91], as well as altering the time that distal *Hoxa13* expression is initiated in digit-forming cells [88,92].

**Figure 2:**
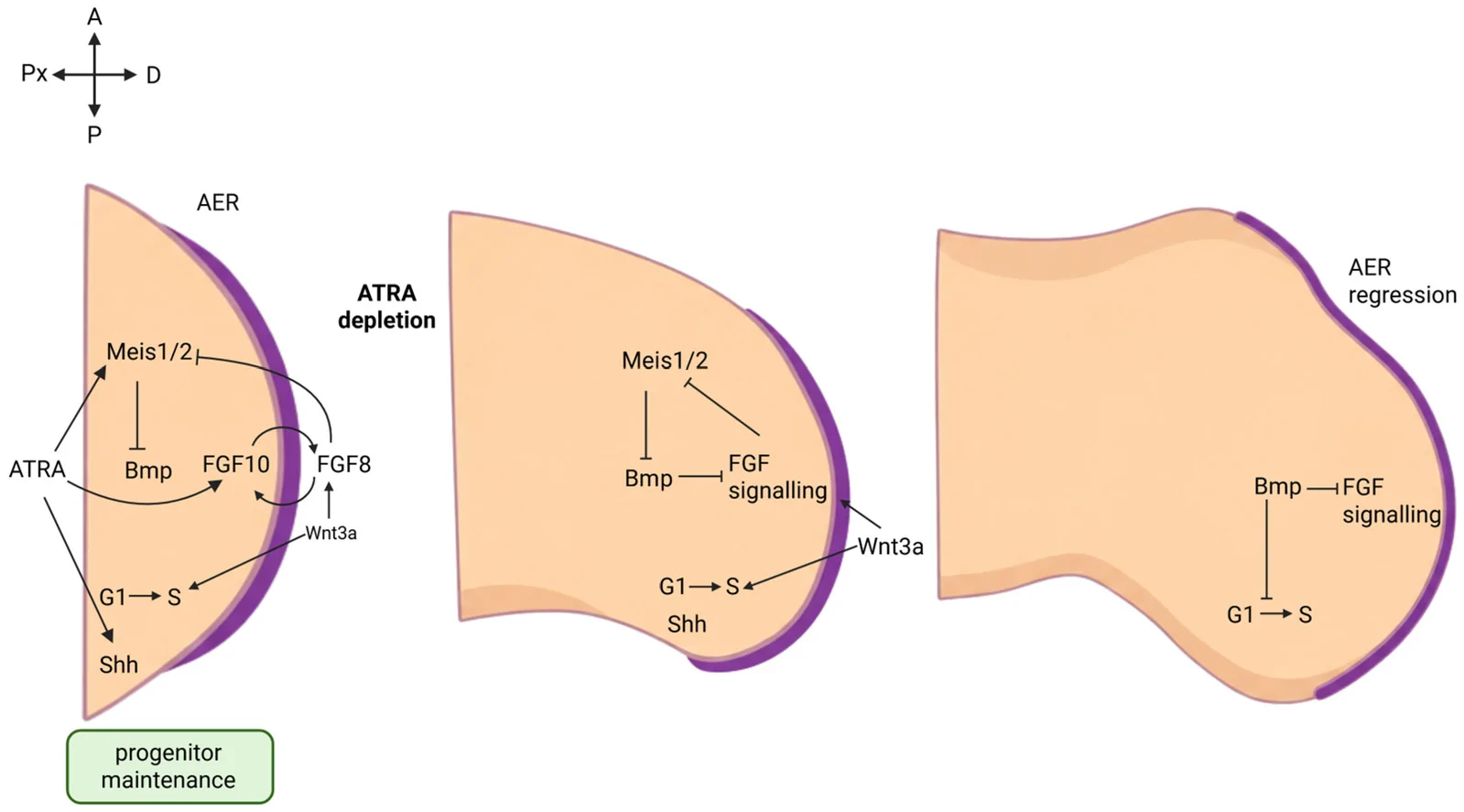
Overview of the temporal progression of the major signaling pathways that regulate chick limb bud initiation, outgrowth, and maturation. **Left** (limb induction and early outgrowth): Proximally derived ATRA is required for limb induction and initiates expression of FGF10 in the lateral plate mesoderm and Shh in the posterior mesenchyme. Reciprocal signaling between mesenchymal FGF10 and apical ectodermal ridge (AER) FGF8, reinforced by Wnt3a, establishes the positive feedback loop that sustains limb bud outgrowth. During this stage, ATRA maintains proximal identity through Meis1/2 expression, promotes maintenance of undifferentiated mesenchymal progenitor cells, and restrains BMP activity. **Middle** (progressive outgrowth): As ATRA becomes depleted from the distal limb bud (approximately HH20/21), the intrinsic distal developmental program is initiated. Wnt3a-dependent FGF signaling continues to support AER function and proliferative outgrowth, whereas BMP signaling progressively restricts FGF signaling and contributes to developmental timing while Shh expression is maintained. **Right** (late outgrowth and growth termination): Continued BMP activity promotes AER regression, suppresses FGF signaling, reduces progression through the G1-to-S phase of the cell cycle, and terminates proliferative outgrowth as the limb transitions towards differentiation and digit patterning. Arrows indicate activation or promotion, blunt-ended lines indicate inhibition, and circular arrows denote the reciprocal positive feedback between FGF10 and FGF8 during early limb outgrowth. Abbreviations: A, anterior; P, posterior; Px, proximal; D, distal; AER, apical ectodermal ridge; ATRA, all-trans retinoic acid; BMP, bone morphogenetic protein; FGF, fibroblast growth factor; Shh, sonic hedgehog; Wnt, wingless/integrated. Created in BioRender. Mcqueen, C. (2026) https://BioRender.com/u1hqeis (accessed on 12 July 2026).

To our knowledge, there are no studies that have directly examined the role of RARγ in chicken limb development. Nonetheless, recombinant limb studies have provided support to the hypothesis that RARγ is responsible for morphogenic patterning [86,92]. The expressions of RARγ and Cyp26b1 overlap in the developing mouse limb, suggesting that RARγ is responsible for ATRA’s teratogenic action. ATRA-induced proximodistal pattern defects were partially rescued by compound knockout of Cyp26^−/−^/Rarγ^−/−^, but these mutants retained zeugopod truncation despite increases in stylopod formation and digit numbers. The double-knockout mutants showed reduced apoptosis, particularly in anterior and posterior necrotic zones, revealing a mechanism by which RARγ may regulate patterning of the limb [93]. The precise role of RARγ during limb development is yet uncertain. However, findings have established the prime importance of ATRA and other cardinal principles. Signaling via Wnts, BMPs, and FGFs coordinate limb initiation, outgrowth, and patterning as summarized in Figure 2. The influence of RARγ on the pathways within stem/progenitor cells is considered below.

## 6. Findings for the Modes of Action of RARγ Within Stem/Progenitor Cells

RARγ regulates retinoid metabolism from comparison of the mRNAs expressed within ATRA-treated RARγ null and wild-type ESCs. The genes regulated included those that encoded STRA6, lecithin-retinol acyltransferase (LRAT, which converts all-trans retinol to retinyl esters), CRABP2, (which transports ATRA within cells to RARs), and the ATRA-catabolizing enzyme Cyp26a1 [94]. RARγ plays a role in reorganizing the *Hox* gene cluster as shown for ESC lines deficient in RARγ [95]. Studies of F9 embryonic teratocarcinoma cells showed that the absence of RARγ led to the loss of ATRA-inducible expression of *Hoxa-1* and *Hoxa-3* [20], and expressions of *Hoxc-11a* and *Hoxb-13a* were lost within the zebrafish embryos treated with the RARγ agonist [40].

Ligand-activated RARγ enhanced the reprogramming of epiblast stem cells into iPSCs. The addition of 0.1 nM ATRA (a concentration that specifically activates RARγ) to an all-trans retinol-free culture medium led to the enhancement of iPCS formation and/or growth, whereas RARγ antagonism (with CD2665) had a deleterious effect [33]. RARγ agonist (CD437) treatment of epiblast stem cells led to enrichment of β-catenin in RARγ immuno-precipitates as probed using an antibody to β-catenin. A weak interaction of β-catenin with RARγ was seen in the absence of agonists. TOPflash experiments, for Wnt signaling, were undertaken to examine crosstalk between RARγ and the Wnt pathway. CHIR99021 increased reporting, which was significantly reduced by RARγ agonism (0.1 nM ATRA and CD437) but not by RARγ antagonism. Hence, physical interaction between liganded RARγ and β-catenin had modulated Wnt signaling in a negative way to promote reprogramming [33]. RARγ and LRH1 agonism enhanced the derivation of iPSCs from human dermal fibroblasts. This was attributed to enhanced TGF-β signaling because transcripts for superfamily members and signaling pathway components were prominent within the iPSCs cells. Additionally, TGF-β substituted for the use of the agonists of RARγ and LRH-1 to induce naive-like pluripotency. When the iPSCs were cultured with the TGF-β signaling inhibitors, SB431542 and A83-01, their numbers declined as cells differentiated. Together, the findings support the view that TGF-β plays a role in sustaining naive-like pluripotency [35].

From studies on the influence of ligand-activated RARγ on bone formation, the investigators concluded that RARγ had blocked the chondrogenic phase of heterotopic ossification [51]. RARγ agonist-treated mesenchymal limb cells were unresponsive in vitro to BMP-2, and skeletogenic potential had either been lost or blocked. For confirmation, ATD5 chondrogenic progenitor cells were transfected with the reporter plasmid Id1-Luc and BMP-2 increased luciferase activity, which was counteracted by the RARγ agonist CD1530. The levels of Smad proteins and phosphorylation of Smad1, Smad5, and Smad8 were decreased. Similarly, BMP-2 is one of the main chondrogenic factors and induces chondrogenic differentiation of various types of stem cells as well as osteogenic differentiation and ossification in mesenchymal stem cells [96]. In contrast, RARγ regulated the expression of BMPs within F9 embryonal carcinoma cells, and induced BMP-2 and reduced BMP-4 expression from the use of RAR-selective ligands [97].

As discussed, the defects seen from the RARγ agonist-treated zebrafish embryos included truncation, loss of fins, craniofacial bones, and anterior neural ganglia, and cardiac edema [40]. Ligand-activated RARγ may have interfered with FGF signaling because 1 nM of the pan-RAR agonist TTNPB, being sufficient for RARγ agonism, downregulated expression of FGF-binding protein. This protein enhanced FGF signaling in ME-180 squamous cell carcinoma cells [98]. Additionally, RARγ agonist treatment of zebrafish embryos provoked defects in zebrafish that collectively mimic those for FGF deficiencies in zebrafish. Axis shortening has been attributed to loss of FGF8a plus FGF24 [99] and loss of pectoral fin formation to FGF16 [100] and FGF24 [101]. Furthermore, FGF signaling was needed to regenerate fins [102]. Heart size and chambers also were affected in FGF8 mutants [103].

FGFs are required for stem/progenitor cell decision-making [104]. They also play roles in regulating cell survival [105] and cell mobility [106], whereby migration is important to pattern formation [107,108]. Mutation of FGF8 impaired craniofacial development via an influence on cranial crest cell migration and survival [109]. FGF24 was needed for the migration of Tbx5-expressing cells to the posterior fin bud [101]. It is noteworthy that RARγb mRNA was expressed from the shield to the 80% epiboly stage during early zebrafish embryogenesis when cell migration is a key feature [110]. Nonetheless, the effects of FGFs are, in part, mediated by interplay with Wnt signaling [111,112], whereby crosstalk patterns the early gastrula [113]. The defects seen for double mutants of disheveled proteins, which mediate Wnt pathway activation, included axis truncation, craniofacial anomalies, skeletal defects, and cardiac edema [114], and RARγ agonism may have interfered with Wnt signaling. It is intriguing that the defects seen for the RARγ agonist-treated zebrafish embryos resemble those for fish deficient in Wnt and fgf signaling. However, a direct mechanism regarding the action of RARγ has yet to exist.

## 7. Ligand-Activated RARγ Regulates a Gateway to Stem/Progenitor Cell Development

Ligand-activated RARγ blocked stem/progenitor cell development for zebrafish embryos, mouse gastruloids and chondrogenesis. Conversely, RARγ antagonism or its absence is conducive to differentiation. Mature myeloid cells increased when RARγ was antagonized in mice and HSCs were fewer, and more mature progenitors were increased in RARγ knockout mice. The blockage of zebrafish pectoral and caudal fin development was reversible by the subsequent addition of the RARγ antagonist. Therefore, RARγ agonism had not caused an irreversible loss of developmental potential within stem/progenitor cells. Instead, the potential remained core to the cells. As a model, we propose that ligand-activated RARγ appears to have acted as a gatekeeper to stem-cell developmental progression during embryogenesis and chondrogenesis by virtue of interference with signals that are required for development. Alternatively, the action of agonized RARγ may have been to ensure the developmental state of stem/progenitor cells per se, including perhaps quiescence. This would also explain the many defects seen for zebrafish embryos treated with the RARγ agonist. In this case, permissibly or otherwise, the generation of mature cells is in a dynamic state and the gene regulatory circuits are dynamic [115,116,117].

RARγ plays a modulatory role because HSCs persisted in the RARγ knockout mouse. Ligand-activated RARγ prevented gastruloid development by virtue of modulation of CHIR99021-provoked and Wnt/β catenin-mediated signaling and the blockage was partially reversible by the RARγ antagonist. Wnt signaling is a core regulator of cell fates [118], and the pattern of defects seen when zebrafish embryos were treated with the RARγ agonist (see above) is compatible with interference with Wnt signaling. Mesenchymal limb cells treated with the RARγ agonist were unable to differentiate in response to BMP-2. This may also be due to a negative influence on Wnt signaling, which activates the downstream targets of BMP [119]. Regarding facilitation of the generation of iPSCs from epiblast cells, agonized RARγ had a negative influence on Wnt signaling, presumably to enhance cell stemness [33]. It is important to note that the influence of RARγ on mature cells was different because non-liganded RARγ dissociated β-catenin from the lymphoid enhancer/T-cell factor within mouse epiphyseal cartilage chondrocytes and agonized RARγ stimulated the expression of Wnt proteins, receptors, and co-receptors [68]. The different findings were from developmental studies versus for cartilage chondrocytes, revealing that the action of RARγ is context-dependent. Nevertheless, RARγ clearly integrates into the Wnt/β-catenin pathway, as also seen from studies of solid tumor progression [reviewed in [120]]. Even so, the regulatory role(s) of RARγ is not yet fully elucidated because 19 Wnts and 10 Frizzled receptors regulate cell fate [121] to trigger cells to either maintain their stem-like state or specialize [122]. A threshold model envisages that Wnt influences are summated regarding an outcome’s influence on cell behavior [123].

**Figure 3:**
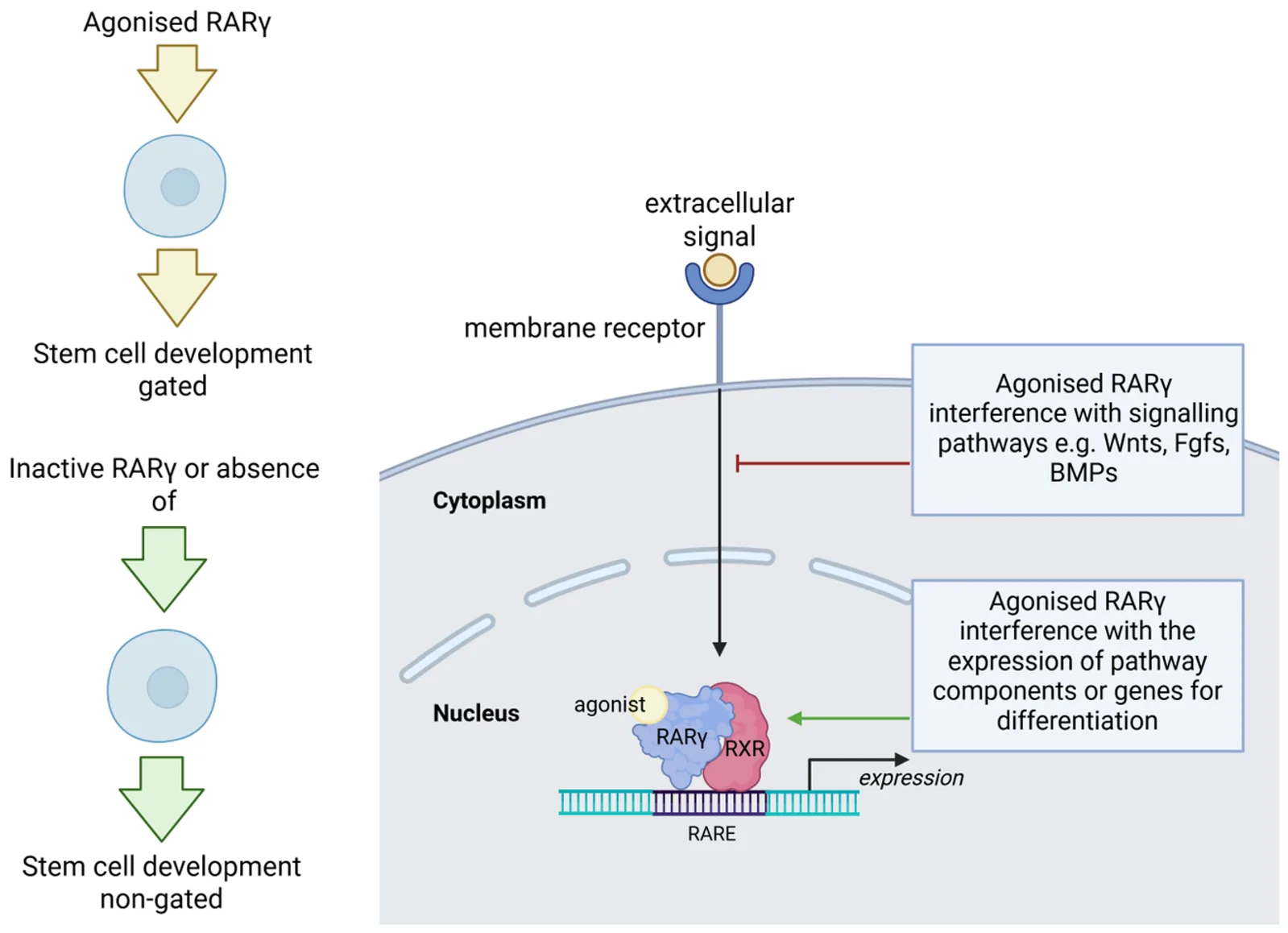
Some principles to the actions of RARγ. Agonism of RAR blocked stem/progenitor cell development, as seen from studies of zebrafish embryogenesis, gastruloid development, and chondrogenesis, and enhanced the generation of iPSCs from somatic cells. RARγ knockout in mice reduced hematopoietic stem cells, and there was an increase in more mature progenitors and mature myeloid cells when RARγ was antagonized in mice. The absence of active RARγ appears to be permissive to differentiation. RARγ plays a role in the nucleus to regulate the expression of genes that are important to the control of stem cell behavior and influences key intracellular signaling pathways. Created in BioRender. Mcqueen, C. (2026) https://BioRender.com/xyzbuxf (accessed on 12 July 2026).

RARγ modulated the expression of TGF-β superfamily members, which are also core regulators of embryogenesis [124]. Agonized RARγ enhanced the expression of TGF-β signaling pathway components regarding the generation of iPSCs from human dermal fibroblasts. TGF-β maintained the ground state pluripotency of human ESCs [125] and chemically reset human cR-H9 naïve pluripotent stem cells [126]. It has also been used with activin, a member of the TGF-β superfamily, to establish human iPSCs [127]. It is interesting to note that the effect of agonizing RARγ within HepG2 hepatocytes was different from that seen for the generation of iPSCs because agonizing blocked the responsiveness of HepG2 cells to TGFβ and antagonism of RARγ rendered cells more responsive to TGFβ. Again, the different findings are for stem cells versus mature cells whereby the action of RARγ is context-dependent. To add to the above, RARγ positively and negatively regulates a very complex network of genes. They include the expression of NOTCH ligands and their receptors, the *Hox* gene cluster, *RARγ*, *PPARγ*, and *RXR*α, and genes pertaining to cell identity, extracellular matrix communication, and ATRA synthesis and catabolism. Figure 3 shows an overview of principles of the actions of ligand-activated RARγ.

**Figure 4:**
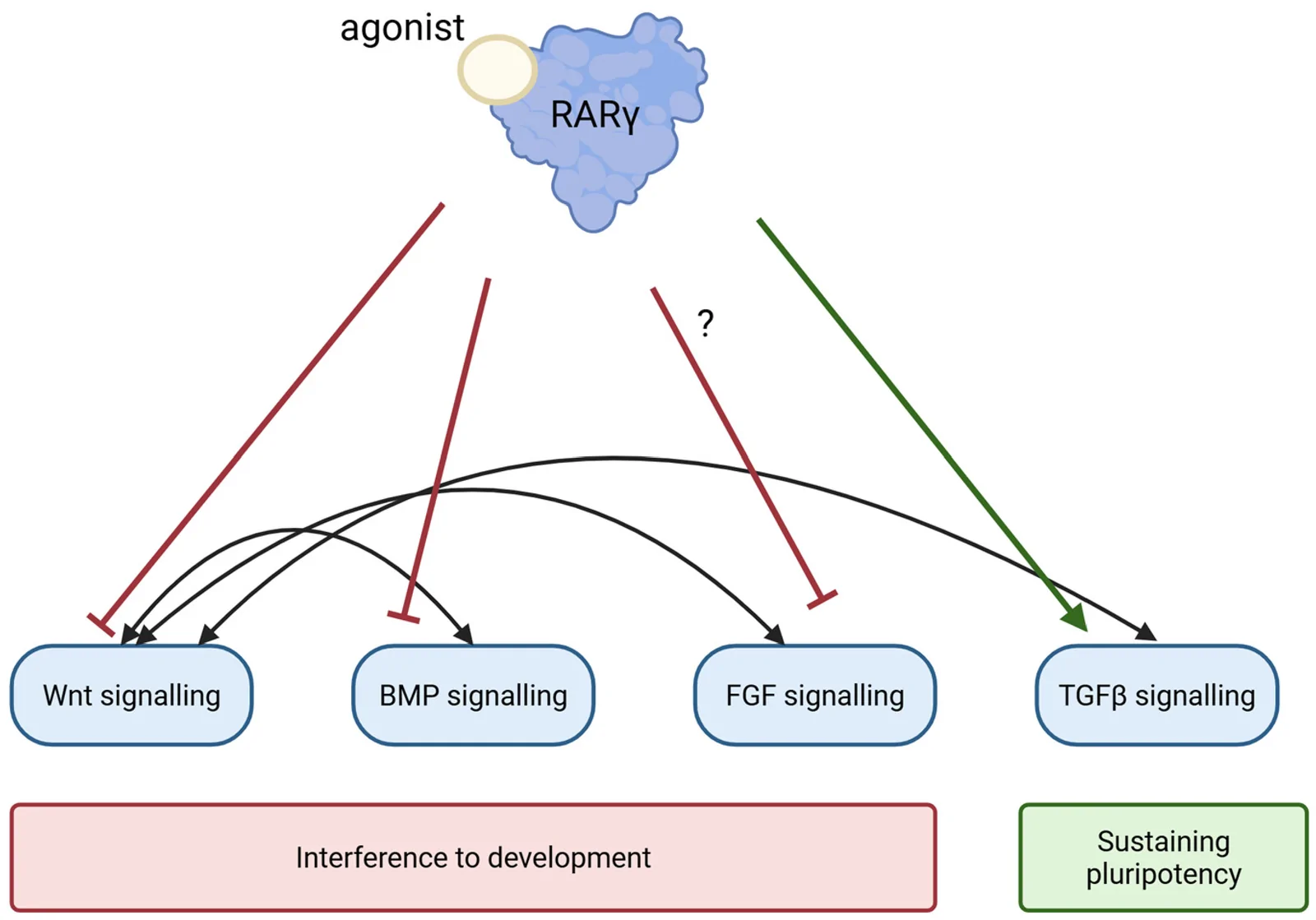
Crosstalk between the regulatory and signaling pathways of interest. For events regulated by agonized RARγ, the red and green arrows indicate a positive and negative influence, respectively, on other pathways. A negative influence (red arrows) was seen for Wnts signaling from studies of the generation of iPSCs, for BMP studies of chondrogenesis, and for FGFs because the RARγ agonist-mediated defects seen during zebrafish embryogenesis mimicked defects seen in FGF mutant zebrafish. A positive effect (green arrow) of agonized RARγ was seen regarding TGFβ signaling and the generation of iPSCs. The double-ended arrows indicate crosstalk between pathways. Created in BioRender. Mcqueen, C. (2026) https://BioRender.com/x71ggtc (accessed on 12 July 2026).

RARγ is largely viewed as a transcriptional regulator. However, RARγ also plays a role within the cytoplasm. This dual functionality may ensure tight control on the developmental progression of stem/progenitor cells. Additionally, the pathways that RARγ integrates with crosstalk with each other. Hence, whether stem/progenitor cells differentiate is regulated by a complex network of signaling events whereby their duration and integration, whether events are cooperative or counteractive, are important [Figure 4]. Crosstalk between Wnt signaling and BMPs during mesenchymal limb development [119,128] is often synergistic, with both pathways contributing to an outcome that each alone cannot achieve. There is crosstalk between Wnts and FGF [111,112] and TGFβ signaling [129,130,131], and a Wnt signaling timing mechanism coordinates ATRA and FGF gradients during development [132]. Figure 4 summarizes the influences of RARγ on some key pathways and that they crosstalk.

Importantly, RARγ and RARα are co-expressed within stem/progenitor cells whereby ligand-activated RARα drives cell differentiation as described for myelopoiesis [133]. A view is that these RARs balance the proper conduct of hematopoiesis by virtue of regulating stemness versus differentiation, respectively [27]. Similarly, RARγ is the predominant RAR within the skin of humans and mice [134,135], with findings indicating that RARα and RARγ play different roles to balance skin homeostasis [136]. As considered above, stem cells do not appear to synthesize ATRA [30,31], nor did the RARγ^+^ stem cells within developing gastruloids [48]. Perhaps transactivation of RARγ by a sub nM level of ATRA guarantees stem-cell maintenance and that transactivation of RARα by a higher level of ATRA is needed for differentiation. Expression of RARγ is lost during myelopoiesis [27], and RARα2 increases dramatically [137]. In this case, progenitor cells that are differentiating towards mature cells would not be subject to a RARγ-mediated restraint to development, with RARα playing a dominant role. Hence, the developmental switch from co-expression of RARγ and RARα to expression of just RARα is important in the conduct of hematopoiesis. RARγ expression within cells is highly dynamic [138] as RARγ has a half-life of 8 h [139], with NTD phosphorylation leading to ubiquitination and degradation [140,141]. The miR-30a-5p rapidly downregulated RARγ protein levels [142], and miR family members regulate organ development [143]. RARγ-mediated transcription is also tightly regulated by vinexin β, which is present in the cytoplasm and nucleus, and interacted with the non-phosphorylated AF-1 domain of RARγ to suppress mediated transcription [144]. Transcriptional activity of RARγ would also be affected by localization to the cytoplasm. Regarding the loss of RARγ, a lack of activity of RARγ, by means of antagonism, increased the responsiveness of HepG2 hepatocytes to an extracellular signal.

Some matters remain uncertain regarding the importance of RARγ in development. RARγ appears to influence the behavior of both stem and progenitor cells. From studies of Xenopus axis elongation, RARγ agonism led to progenitor cell death to terminate elongation, thus influencing tissue development. Might the dual roles of stem and progenitor cell behavior relate to the nuclear-versus-cytoplasmic compartmentalization of RARγ, where it is required for the formation of the Riptosome complex for cell death? RARγ has been implicated in cancer initiation [120] and in aggressive and metastatic disease regarding expression with cancer stem cells [67]. A precise mode of action in either case is still unclear. Overexpression of RARγ not only enhances cell proliferation [reviewed in [145]] but may also shift the balance in the behavior of cancer cells in favor of stemness, contributing to the often-impaired maturation of cancer cells. Nonetheless, RARγ plays a pivotal role by virtue of interacting with pathways that are the cornerstones of many cancers, namely Ras-, p53-, and Myc-mediated perturbations. Ras and the CPF6-RARγ fusion protein play a synergistic role in aggressive acute myeloid leukemia [146]. The use of acacetin to displace ATRA from RARγ switched AKT-p53 from a pro-survival to pro-apoptosis role [147]; there is an interplay between RARγ and Myc in breast cancer [148], and RARγ binds to the Myc promoter in pancreatic cancer [14].

## 8. Concluding Remarks

During embryogenesis, agonism of RARγ prevented the development of stem/progenitor cells. Agonism also promoted the generation of iPSCs from somatic cells. There is evidence to support the converse that non-liganded RARγ is permissive for development. In this case, RARγ guards the transition of stem cells from non-permissive to a permissive state for differentiation pending its ATRA ligation status. This role of RARγ also extends to stem/progenitor cell homeostasis within adult tissues. The events that are regulated by RARγ play crucial roles in governing stem and progenitor cell behavior, including the expression of transcription factors, modulation of the responsiveness to extracellular factors, and an influence on intracellular signaling pathways. Understanding the roles of RARγ has largely focused on the direct expression and suppression of genes. However, the actions of RARγ are more complex, including its role as a co-factor to other nuclear regulators and within the cytoplasm.

RARγ plays a role in the maintenance of stem cells, as seen from developmental studies, and in the generation of iPSC. The ATRA ligation status of RARγ may be regarded as a gatekeeper to stem/progenitor cell developmental progression. From studies on cancer cells, RARγ has a role to play in cell proliferation by virtue of its overexpression contributing to the enhancement of proliferation. Major knowledge gaps are the extent to which these two roles are pivotal to the maintenance of stem cells and the aggressive nature of some cancers. Also, there is the need for future studies to identify the associated key regulatory events.

## Data Availability

No new data were created or analyzed in this study. Data sharing is not applicable to this article.
